# Severe hypernatremia in children after surgical resection of hepatic echinococcosis: a rare and potentially fatal complication

**DOI:** 10.1186/s12887-021-02607-1

**Published:** 2021-03-24

**Authors:** Kewei Li, Yijun Liu, Xiaolong Xie, Rongxing Zhou, Bo Xiang

**Affiliations:** 1 Department of Pediatric Surgery, West China Hospital of Sichuan University, 610041 Chengdu, China; 2 West China School of Medicine of Sichuan University, Chengdu, China; 3 Department of biliary surgery, West China Hospital of Sichuan University, Chengdu, China

**Keywords:** Hypernatremia, Children, Liver resection, Echinococcosis

## Abstract

**Background:**

Using effective scolicidal agents intraoperatively is essential to lessen the recurrence rate of hepatic echinococcosis. However, severe hypernatremia may occur after hypertonic saline (HS) has been applied as the scolicidal agent. The aim of this study is to report on pediatric patients with severe hypernatremia after hepatic echinococcus surgery.

**Methods:**

Patients who presented to West China Hospital between January 2010 and February 2017 were retrospectively analyzed. Children under 16 years with echinococcosis treated by resection were included in the study.

**Results:**

A total of 26 children were enrolled in this study, including 16 boys and 10 girls with a median age of 8 (2–16). 24 (92.3 %) cases were cystic echinococcosis (CE) and two (7.7 %) were alveolar echinococcosis (AE). According to Clavien-Dindo classification of surgical complications, the complication rate of all 26 patients was 19.2 %, among which three cases belonged to Grade I, one to Grade III b and 1 to Grade IV. Two children encountered severe hypernatremia (sodium: 155.3 mmol/L and 190.0mmol/L). Data showed classic clinical features of severe hypernatremia: profound and persistent bradycardia, hypotension and coma. After treatment, they recovered well without any neurologic sequelae. All patients were followed up regularly for a median time of 38 months (range 4–89 months); the overall disease-free survival was 100.0 %.

**Conclusions:**

HS irrigation of intra-abdominal echinococcosis may cause acute hypernatremia and severe consequences. Diagnostic suspicion and early intervention are vital tools for avoiding morbidity and mortality.

## Background

Echinococcosis is a severe helminthic zoonosis caused by the adult or larval tapeworm of Echinococcus (E) granulosus, E. mulitilocularis, E. vogeli or E. oligarthrus; it most frequently involves the liver (50–70 %) [1]. The two major species of medical and public health significance that bring about hepatic echinococcosis are E. granulosus and E. multilocularis, which cause cystic echinococcosis (CE) and alveolar echinococcosis (AE), respectively. AE is mainly distributed in the northern hemisphere, while CE prevails worldwide [2].

Surgery is the main mode of treatment for these diseases. Recurrence during a long-term follow-up is the primary problem with this treatment option. Using effective scolicidal agents intraoperatively is essential to lessen the recurrence rate in patients with spillage of cyst contents [3, 4]. Hypertonic saline (HS) is one of the most common scolicidal agents to prevent recurrence on a global scale. HS has been recommended by WHO/OIE as a scolicidal agent in various concentrations (15–20 %) with exposure time of at least 15 min^2^. Iatrogenic hypernatremia, defined by a serum sodium concentration of more than 145 mmol/L, although rare, can occur after HS has been applied as the scolicidal agent of echinococcus [5]. It is noteworthy that hypernatremia is highly associated with mortality (40–60 %) [6] and is particularly fatal in cases in which hypernatremia occurs very rapidly. Here, we report on pediatric patients with severe hypernatremia after hepatic echinococcus surgery, who were treated in the pediatric intensive care unit.

## Methods

### Patients

Between January 2010 and February 2017, patients with echinococcosis treated with operative resection were retrospectively analyzed. The data were collected from patient charts and electronic medical records of the patients with echinococcosis (ICD-10 code B67.5 and B67.8) at West China Hospital of Sichuan University. We included the patients who were diagnosed with echinococcosis from birth to 16 years of age and received resection as a treatment. Our study did not enroll transplantation cases. Written informed consent was obtained from all patients’ parents. The data collected included demographic data (sex, age and children from pastoral area), symptoms (vomiting, abdominal pain, diarrhea, distention, constipation, and duration of symptoms), signs (temperature, palpable mass, and location of the mass) and imaging and laboratory data (ultrasonic examination, CT/MRI/MRCP and echinococcus enzyme linked immunosorbent assay (ELISA)). Clinical records were independently reviewed by two authors.

### Treatment regimen

The surgical options included endocystectomy, non-anatomical liver resection, and intra-luminal cholangiohydatid resection. A subcostal incision was employed to ensure adequate exposure of the liver. During the operation, copious viscous fluid and colloid cyst contents were found after lancing the pericystic membrane in most CE cases. After aspiration of the cyst fluid, 20 % HS was injected into the cyst cavity and incubated for 5 min. The HS and cyst contents were then carefully removed, followed by irrigation of 20 % HS into the cyst cavity for 10 min and, ultimately, normal saline irrigation. For certain stages of CE and AE with cystic features, hepatectomy is a necessary approach. All diagnoses were confirmed by histopathological examination of the cases. Hypernatremia was defined as serum sodium level exceeding 145mmol/L.

### Statistical analysis

All results were expressed as the mean values ± standard deviations or medians as appropriate. Statistical analysis SPSS software (version 24.0, SPSS Company, Chicago, IL) was used to perform all statistical analyses.

## Results

### Patient characteristics

A total of 26 children were enrolled in this study, including 16 boys and 10 girls with a median age of 8 (2–16). Characteristics of the study population are described in Table 1. Twenty-five (96.2 %) patients were from pastoral areas. Of the children included in this study, eight (30.8 %) had no clinical symptoms, 18 (69.2 %) had mild symptoms (including abdominal pain and discomfort) and none had severe symptoms. Twenty-one (80.8 %) patients had positive results for the echinococcus ELISA test. Based on ultrasound liver images, the WHO classification [7] of lesions included 20 (76.9 %) patients belonging to CL/CE1/CE2, four (15.4 %) patients belonging to CE3 and two (7.7 %) patients belonging to CE4/CE5. The surgical procedures included endocystectomy in 14 patients, endocystectomy with non-anatomical liver resection in five patients, non-anatomical liver resection in four patients, intra-luminal bile duct mass resection in one patient and endocystectomy in both liver and right lung in two patients. According to constellation of histology findings, 24 (92.3 %) cases were CE and two (7.7 %) were AE.

**Table 1:** Patient characteristics and histopathological features

Variables	Results
Sex (male/female)	16/10
Age (y)	2–16
Children from pastoral area	25(96.2 %)
Children with abdominal symptoms	18(69.2 %)
Echinococcus ELISA positive	21(80.8 %)
WHO classification of ultrasound images	
CL、CE1、CE2	20(76.9 %)
CE3	4(15.4 %)
CE4、CE5	2(7.7 %)
Surgical procedure	
Endocystectomy	14(53.8 %)
Endocystectomy with non-anatomical hepatectomy	5(19.2 %)
Non-anatomical hepatectomy	4(15.4 %)
Intra-luminal bile duct mass resection	1(3.8 %)
Hepatopulmonary endocystectomy	2(7.7 %)
Histopathological feature	
E. granulosus	24(92.3 %)
E. multilocularis	2(7.7 %)

*ELISA*  enzyme linked immunosorbent assay

### Postoperative data

**Fig. 1:**
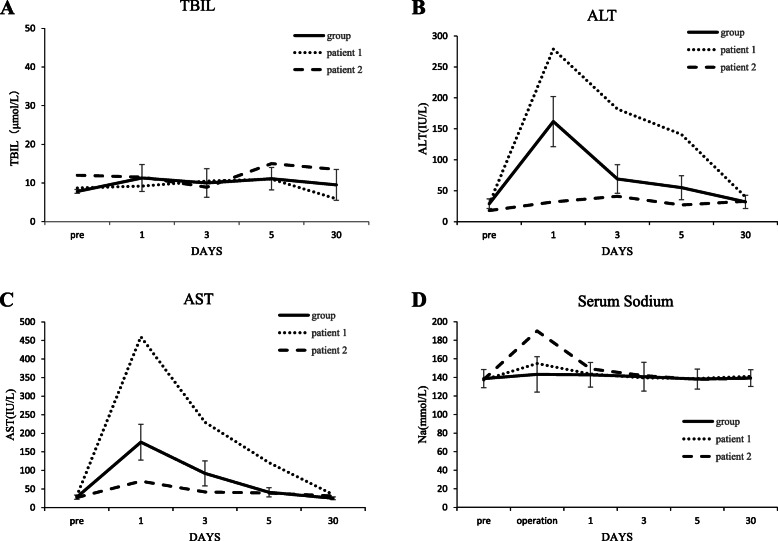
Biochemical analysis of 26 children with hepatic echinococcosis. **a** Changes in blood total bilirubin (TBIL) values during the hospital stay and follow-up. The results of whole cohort, and patient 1 and 2 are displayed separately. No obvious abnormality was observed. **b** and **c** Aminotransferase (ALT and AST) analysis. Mild increase with a peak on postoperative day (POD) 1 was observed. **d** In most children, serum sodium level was within normal limits; however, two children (patients 1 and 2) had severe hypernatremia at the end of surgery and returned to normal levels only after intervention

Table 2 presents postoperative results. Ten surgical complications occurred in five patients, including three bile leakages, one episode of bleeding, one unilateral pleural effusion, one ascites, one bronchopleural fistula, two cases of hypernatremia and one reoperation due to intestinal obstruction at one month postoperatively. In addition, temporary elevation of serum transaminases was recorded in 10 patients. The mean serum level of alanine aminotransferase (ALT) and aspartate aminotransferase (AST) were 161.8 ± 40.6 IU/L and 176.1 ± 48.3 IU/L, respectively (Fig. 1. a, b and c). According to the Clavien-Dindo classification of surgical complications, the complication rate of all 26 patients was 19.2 %, among which three cases belonged to Grade I, one to Grade III b and one to Grade IV. A median pediatric intensive care unit (PICU) stay of 12.7 (range 0-120) hrs was required, and the mean post-operative length of hospital stay (LOS) was 8.3 ± 3.6 days. All patients were followed up regularly for a median time of 38 months (range 4–89 months); the overall disease-free survival was 100.0 %.

**Table 2:** Postoperative data of patients

Variables	n (%) / Results	
Bile leakage	3(11.5)	
Postoperative hemorrhage	1(3.8)	
Pleural effusion	1(3.8)	
Ascites	1(3.8)	
Bronchopleural fistula	1(3.8)	
Elevation of serum transaminase (ALT vs. AST IU/L)	10(38.5) / 161.8 ± 40.6 vs. 176.1 ± 48.3	
Hypernatremia(mmol/L)	2(7.7) / 155.3 vs. 190.0	
Reoperation	1(3.8)	
Clavien-Dindo classification		
Grade I	3(11.5)	
Grade II	0(0.0)	
Grade IIIa	0(0.0)	
Grade IIIb	1(3.8)	
Grade IV	1(3.8)	
Grade V	0(0.0)	
Hospital stay(d)	8.3 ± 3.6	
Follow-up(m)	38(4–89)	
Disease free survival	26(100)	

*ALT* alanine aminotransferase; *AST* aspartate aminotransferase

### Data of two children with severe postoperative hypernatremia

The clinical characteristics of two children with hypernatremia are shown in Table 3. Patient 1 was a 5-year-old boy suffering from abdominal pain. He was admitted to hospital for removal of multiple hydatid cysts (CE2m, by WHO classification) with a maximum diameter of 8.6 cm. Endocystectomy and partial liver resection were performed, and the following histologic examination indicated the involvement of E. granulosus. After aspiration of the cysts’ contents, a total capacity of 500 mL 20 % HS was injected into each cyst cavity and left for 15 min, respectively. The fluid was then cautiously removed by an aspirator. The patient subsequently experienced skin rashes, hypotension, anaphylactic shock and hypernatremia (sodium: 155.3 mmol/L) (Fig. 1.d) at the end of operation. Hypotonic fluid and 5 % dextrose were infused intravenously. Shortly afterwards, the patient was transferred to the PICU with an endotracheal tube in place. The serum sodium decreased to a normal range on postoperative day (POD) 1, then successful extubation was performed. The patient experienced mild ascites for the next few days. Approximately eight days after surgery, the patient made a swift recovery and was well at follow-up.

**Table 3:** Data of 2 patients with postoperative hypernatremia

Variables	Patient 1	Patient 2
Age (y)	5	11
Sex	M	F
Number of cysts	Multiple	2
Maximum diameter of Cyst(cm)	8.6	10
WHO classification	CE2m	CE2m
Surgical procedure	Endocystectomy and partial liver resection	Endocystectomy
Histopathological feature	E. granulosus	E. granulosus
Peak serum sodium (mmol/L)	155.3	190
Blood transfusion	0	0
Complication	Mild ascites	Bile leakage and bronchopleural fistula
PICU stay (h)	18	120
Postoperative hospital stay (d)	8	19

*PICU* Pediatric intensive care unit

Patient 2 was an 11-year-old girl with abdominal distension severe enough to warrant hospital admission, diagnosed as hepatopulmonary hydatid cysts (CE2m, by WHO classification) with a maximum diameter of 10 cm, located on the liver and superior lobe of the right lung. The patient underwent endocystectomy and CE was then confirmed by thorough histologic examination. After aspiration of the cysts’ contents, a total capacity of 800 mL 20 % HS was injected into each cyst cavity and left for 15 min. The patient experienced a delayed recovery after general anesthesia, suffering from postoperative hypernatremia (sodium: 190.0mmol/L) (Fig. 1.d), and immediately underwent tracheal intubation and was transferred to the PICU. Hypotonic fluid and 5 % dextrose were administered intravenously, and sodium decreased to a normal range on POD 2. Successful extubation was performed on POD 2. However, the patient developed bile leakage and bronchopleural fistula. On POD 3, the patient became unresponsive and bradycardic (heart rate: 55–60 beats per minute), although all serum electrolytes were within normal limits at that time. She was re-intubated for one day. Successful re-extubation was performed on POD 4, and afterwards, the patient returned to general recovery ward on POD 5. After biliary and thoracic drainage, the patient recovered well and was discharged from hospital 19 days postoperatively without any neurologic sequelae.

## Discussion

Although no clinical trial exists to compare the safety and effectiveness of all the various therapeutic protocols in the management of liver hydatid disease, including surgical resection, percutaneous puncture, drug therapy and employment of a watch-and-wait surveillance policy, surgical resection accounts for the most common treatment approach, particularly in patients with large cysts, multiple daughter vesicles or treatment complexity [8].

Given the risk of death and obliterative cholangitis induced by formalin and biliary epithelium injury following the use of silver nitrate, HS is one of the most common and essential scolicidal agents in the world to prevent inadvertent dissemination and avoid recurrence, and is the agent recommended by the WHO/OIE [5]. The effect of HS is attributed to lysis in response to sufficiently strong osmotic gradient across the outer cuticular membrane of the scolex [4]. Although HS has been used in various concentrations (3–30 %) for various exposure regimens (5–30 min) according to reports in the literature, and recommended by the WHO/OIE in various concentrations (15–20 %) using an exposure time of at least 15 min, there is no clear consensus on the optimal concentration or exposure time as far as scolicidal effect is concerned [4].

Iatrogenic hypernatremia is routinely caused by the administration of large doses of sodium bicarbonate (e.g. during a circulatory arrest or intoxication) and HS perfusion (e.g. during stomach lavage, artificial abortion or echinococcosis cyst irrigation); hypernatremia can at times be the result of ingesting large amount of high-salt diet [9–12].

The mechanism of hypernatremia after a HS injection during surgery of echinococcosis may involve: absorption through the cyst wall, absorption from the digestive tract via a cyst communication with the biliary ducts (suggested by bile-stained cyst contents), spillage of HS in the peritoneum or pleura, or unwitting injection of HS into hepatic blood vessels [13, 14]. In our study, hypernatremia might have resulted from absorption of HS through cyst walls, and via the exchange activity of both salt and water across the peritoneal membrane and pleura.

Life-threatening hypernatremia causes plasma water volume expansion and intracellular water volume reduction; hence it causes cerebral dehydration and pulmonary edema with convulsions and coma as clinically critical sequelae [8]. Data from our patients showed classic clinical features of severe hypernatremia: profound and persistent bradycardia, hypotension and coma [15]. As mentioned in the literature, among patients who had sodium concentrations above 160 mmol/L for various causes, the mortality rate was 70 %; children tolerated more severe hypernatremia and had better prognoses compared to adults [8]. Few patients in the literature have experienced and survived extreme hypernatremia with serum sodium level greater than 200 mmol/L; most of them were children [16, 17].

Although severe hypernatremia was reported in the English literature in only a few cases after HS therapy, mortality has occurred in at least three patients thus far (see Table 4) [5, 8, 13], including one child reported by Krige et al. This 7-year-old boy died from iatrogenic hypernatremia with a serum sodium level of 170 mmol/L after hepatic hydatid surgery. Among these deaths, two adult patients underwent laparoscopic endocystectomy / partial cystectomy. Diego Anta et al. observed that laparoscopic technique may cause severe or fatal hypernatremia as frequently or more frequently than open operation [18]. Some studies concluded that increased intra-abdominal pressure increases distribution and diffusion of intraperitoneal chemotherapy (with oxaliplatin and cisplatin) in the visceral and parietal peritoneum of an experimental animal model [19, 20]. This constitutes a potential mechanism for fatal hypernatremia in laparoscopic cases. However, no study directly addresses the use of HS under elevated intraabdominal pressure.

**Table 4:** World mortality reports of Echinococcosis with postoperative hypernatremia

Authors	Year	Region	Age(y)	Sex	Surgical type	Saline concentration	Volume (ml)	Exposure time (min)	Peak serum Na (mmol/L)	Postoperative survival
Krige et al. [13]	2002	South africa	7	M	E	20 %	250	5 vs. 3	170	20 h
Michalodimitrakis et al. [8]	2011	Greece	38	F	LPC	15 %	4000	NA	196	3w
Zeng et al. [5]	2017	China	28	F	LE	20 %	300	10	188.8	5d

*Na* sodium; *E* endocystectomy; *LPC* laparoscopic partial cystectomy; *LE* laparoscopic endocystectomy

Acute hypernatremia should be treated more carefully. However, optimal correction rate of acute hypernatremia in children has not been established. Based on experimental data on cerebral volume regulation, a faster pace of correction, such as decreasing serum sodium concentration by 1 mmol/l/h, as we practiced, was suggested for hypernatremia of rapid onset [21, 22]. Even after an intraoperative upward serum sodium shift of 60 mmol/l, a prompt and rational correction of hypernatremia may be in alignment with survival without neurologic sequelae and improve the patient prognosis [21].

Our experience over 26 pediatric cases of operative treatment of echinococcosis indicated that the HS irrigation during operation of echinococcosis may cause hypernatremia with a potentially fatal outcome. Encouragingly, our rapid therapeutic interventions were successful, and mortality was avoided. To our knowledge, this is the first retrospective study on severe hypernatremia in children after surgical resection of hepatic echinococcosis. Our study is inevitably limited by small sample size and lack of a control group. More rigorous experimental protocols should be designed to offer opportunities for prevention and treatment.

## Conclusions

HS irrigation of intra-abdominal echinococcosis may cause acute hypernatremia leading to morbidity and mortality. Diagnostic suspicion and early intervention in the PICU are vital tools for avoiding devastating clinical outcomes. Further studies are needed to achieve a better understanding of postoperative hypernatremia in pediatric patients.

## Data Availability

The datasets are available from the corresponding author on reasonable request.

## Abbreviations

E: Echinococcus
CE: Cystic echinococcosis
AE: Alveolar echinococcosis
HS: Hypertonic saline
ELISA: Enzyme linked immunosorbent assay
ALT: Alanine aminotransferase
AST: Aspartate aminotransferase
PICU: Pediatric intensive care unit
POD: Postoperative day
Na: Sodium
LPC: Laparoscopic partial cystectomy
LE: Laparoscopic endocystectomy
